# Randomized controlled trial of PERMA-based intervention on subjective well-being and post-traumatic growth in young and middle-aged stroke survivors during rehabilitation

**DOI:** 10.1038/s41598-026-54152-5

**Published:** 2026-05-22

**Authors:** Zixiu Zheng, Ruixia Zhang, Jing Kang, Hongxia Lv, Yunxiao Zhao, Zhaohe Song, Yi Yang, Xinyu Mi, Yanqing Wang, Cong Yu

**Affiliations:** 1 Department of Geriatrics, Inner Mongolia Baogang Hospital, Baotou, China; 2 School of Nursing, Chengdu University of Traditional Chinese Medicine, Chengdu, China; 3 Shaanxi Provincial People’s Hospital Neurosurgery, Xi’an, China; 4 Department of Nursing, Shenzhen Second People’s Hospital, Shenzhen, China

**Keywords:** Stroke, PERMA model, Psychological intervention, Young and middle-aged, Subjective well-being (SWB), Post traumatic growth (PTG), Diseases, Health care, Medical research, Neurology, Neuroscience, Psychology, Psychology

## Abstract

Young and middle-aged stroke patients face unique rehabilitation-related psychological distress—such as frustration from slow motor function recovery, anxiety about losing family/social roles, and low motivation for long-term rehabilitation—all of which directly hinder physical recovery and post-stroke adaptation. This study developed a rehabilitation-aligned PERMA (positive emotions, engagement, relationships, meaning, achievement) intervention—rooted in positive psychology and tailored to stroke-specific needs—and evaluated its efficacy in improving subjective well-being (SWB) and post-traumatic growth (PTG) in young and middle-aged stroke patients. A total of 132 eligible patients were randomized into two groups (66 per group) via 1:1 simple randomization, using a computer-generated random number table with allocation concealment via sealed envelopes. The control group received conventional rehabilitation, while the intervention group supplemented with 8 structured PERMA-based sessions over 4 weeks. SWB and PTG were assessed at baseline, post-intervention, 1-month, and 3-month follow-ups. Statistical analyses included independent samples t-tests (between-group comparisons) and repeated-measures ANOVAs (longitudinal changes). SWB scores were significantly higher in the intervention group than in the control group at post-intervention, 1-month, and 3-month follow-ups (t = 2.584, 5.572, 6.099; *P* < 0.05); PTG scores were also significantly higher at these time points (t = 2.750, 3.948, 5.300; *P* < 0.01). Repeated-measures ANOVAs confirmed statistically significant time-dependent increases in both outcomes for the intervention group (*P* < 0.01), with no such significant trend in the control group. The rehabilitation-aligned PERMA intervention effectively enhances SWB and PTG in young and middle-aged stroke patients during recovery. By integrating positive psychology with functional rehabilitation goals, it addresses the unique psychological needs of this population and provides a feasible, clinically applicable support strategy that complements physical rehabilitation.

*Trial Registration*: Registration number of China Clinical Trials Registration Center: ChiCTR2200060103, First Posted date: 18/05/2022, Date of first participant enrollment: 01/12/2022.

## Introduction

According to the latest data from the Global Burden of Disease (GBD) study, stroke is the second leading cause of death among noncommunicable diseases worldwide and the third leading cause of disability-adjusted life years globally^1^. Furthermore, stroke incidence is showing a trend toward younger age groups worldwide^2,3^; it is the primary cause of disability among middle-aged and young adults^4^. Young and middle-aged adults bear significant family and social responsibilities. Despite actively engaging in rehabilitation training, their recovery outcomes remain limited and the process protracted, leading to a substantial decline in well-being^5^. They exhibit lower levels of post-traumatic growth and require a high degree of support^6^.

Psychological issues among stroke patients have drawn scholarly attention. Luo et al.^7^ conducted a PERMA-based intervention on stroke patients aged 18 and above (mean age 66.66 years), which reduced patients’ fear of recurrence and enhanced their psychological capital. Young and middle-aged patients differ from elderly patients. As primary economic pillars and core workforce members in their families, they face unique psychological challenges such as perceived failure and career disruption. Additionally, neurological recovery during rehabilitation requires significant time. Enhancing positive emotions and coping abilities during this period is crucial for improving rehabilitation compliance and exercise efficacy.

Subjective well-being (SWB), a core construct in positive psychology, refers to an individual’s positive evaluation of life quality based on personal criteria—for young and middle-aged stroke patients, this is specifically linked to functional recovery, independent daily living, and reintegration into family/social roles^8^. Studies show 76.58% of stroke patients have moderate-to-low SWB, primarily driven by frustration with rehabilitation setbacks and fear of long-term disability^9^. Guiding these patients to recognize their residual strengths and leveraging positive emotions to address rehabilitation-related distress is key to improving SWB, which in turn promotes adherence to rehabilitation exercises and social participation.

Middle-aged and young stroke patients in the recovery phase commonly experience a lack of positive emotions and unmet psychological needs. Negative emotions are closely linked to their quality of life, and patients demonstrate insufficient coping abilities following their traumatic experience^10^. Tedeschi et al.^11^ introduced the term “post traumatic growth (PTG)” and defined it as positive psychological changes experienced after struggling with traumatic events. A growing body of research has shown that beyond negative psychological responses to stressful events, individuals can also experience positive psychological changes—defined as post-traumatic growth^12^. A longitudinal qualitative study has confirmed that stroke patients can undergo PTG^13^, but young and middle-aged patients often show lower PTG levels due to unmet psychological needs during rehabilitation^14^. Previous studies have shown that positive psychological changes not only complement physical recovery but also modify health behaviors^15^ and facilitate the transition from hospital to home life^16^. Therefore, during the critical phase of rehabilitation, encouraging patients to adopt a positive attitude toward traumatic events and increasing their motivation for recovery plays a crucial role in enhancing rehabilitation outcomes.

However, there is a paucity of interventional research focusing on the rehabilitation-specific psychological needs of young and middle-aged stroke patients. Most existing psychological interventions for stroke patients are deficit-focused (e.g., addressing depression/anxiety), while neglecting the cultivation of positive traits conducive to long-term recovery. “Originating from positive psychology, the PERMA model^17^ was adapted in this study to address core issues during the rehabilitation phase of young and middle-aged stroke patients. Precision in rehabilitation support was achieved through targeted customization of its five key elements: Positive emotions (P) tied to rehabilitation milestones (e.g., joy from independent walking), Engagement (E) in functional recovery activities (e.g., ‘flow’ during hand function training), Relationships (R) with family or recovered peers (e.g., mutual support in rehabilitation), Meaning (M) in post-stroke life (e.g., redefining self-worth beyond pre-stroke roles), and Achievement (A) in functional gains (e.g., mastering self-care skills)^18^”.

PERMA-based psychological interventions have demonstrated positive outcomes among cancer patients ^19^, healthcare workers^19,20^, college students^22^, and psychiatric patients^23^. However, research on their adaptability and application during the rehabilitation phase of young and middle-aged stroke patients remains limited. This study explores the development of a PERMA intervention program for stroke patients in the rehabilitation phase. By stimulating positive emotions and awakening intrinsic motivation for recovery, the program aims to enhance engagement in rehabilitation exercises, increase subjective well-being and post-traumatic growth, and improve coping abilities. This study objectives to develop a one-on-one, rehabilitation-aligned PERMA intervention for young and middle-aged stroke patients (18–64 years) in the rehabilitation phase, and evaluate its efficacy in improving SWB and PTG, with a 3-month follow-up to verify the sustainability of the intervention effects.

## Research subjects and methods

### Research subjects

This study employed a single-blind randomized controlled trial design, with participants and outcome assessors serving as the blinded subjects. Enrolling young and middle-aged stroke patients hospitalized in the Department of Rehabilitation Medicine at a Grade III Class A hospital in Shenzhen, Guangdong Province, between December 1, 2022, and June 30, 2023. *Inclusion criteria*: patients who also met the following conditions: (1) age ≥ 18 years and < 65 years, according to the Chinese expert consensus, this age group is defined as “young and middle-aged^24^; (2) diagnosed with ischemic stroke and meeting the 2019 Chinese Major Cerebrovascular Disease Diagnostic Criteria^25^, ischemic stroke was selected due to its higher prevalence in this age group compared to hemorrhagic stroke, ensuring sample homogeneity; (3) stable disease, to ensure patients could safely participate in the 4-week intervention; (4) modified Rankin Scale (mRS) score ≥ 3^26^ (Patients with moderate to severe functional impairment), Patients with moderate to severe cases may face more significant psychological challenges; (5) normal cognition (Montreal Cognitive Assessment score ≥ 26), this is to ensure that patients understand the intervention, engage in self-reflection, and reliably complete the self-administered questionnaire; (6) informed consent to participate in this study. *Exclusion criteria*: Patients with one of the following conditions (1) patients with other major concomitant diseases, such as heart failure, respiratory failure, malignancy, severe trauma, and other critical illnesses; (2) patients who are participating in other studies. *Withdrawal criteria*: patients with one of the following conditions: (1) patients who were unable to participate in the subject study due to disease regression; (2) patients who voluntarily requested to withdraw from the study due to personal reasons; (3) patients who were lost to follow-up.

This clinical trial was registered at the Chinese Clinical Trial Registry (ChiCTR2200060103, First Posted date: 18/05/2022).

Grouping was performed using a computer-generated random number table method: a statistician not involved in the study implementation generated a random sequence. Patients were sequentially assigned to either the control group or the intervention group based on their admission order. Allocation was concealed using sealed envelopes, ensuring that neither the researchers, patients, nor evaluators were aware of the group assignments.

### Research methods

#### Research team

All members of the research team received uniform training and a division of responsibilities. There was one researcher mentor (a nursing expert in the field of chronic diseases), one national level 2 psychological counselor, two rehabilitation specialist nurses (intermediate level or above), one rehabilitation physician (intermediate level or above), one rehabilitation therapist, and two graduate students in the program. The supervisor was responsible for the overall design of the program and quality control; the counselor was involved in the program design and training of the guiding language; the rehabilitation physician was responsible for the assessment of the disease; the rehabilitation therapist was responsible for the rehabilitation guidance; the rehabilitation specialist nurse was responsible for the routine care; and the two postgraduate students were responsible for the implementation of the program, including sending and receiving questionnaires, data statistical analysis, and thesis writing with the assistance of other members of the research team.

All researchers received the following training: Theoretical training covering the PERMA model of positive psychology, rehabilitation training knowledge for stroke patients, and ethical guidelines; Intervention protocol training involving eight sessions based on the designated research protocol, including communication techniques, training methods, and emotional coping strategies, with role-playing exercises to confirm proficiency in the intervention protocol; Assessment training, familiarization with the Subjective Well-Being Scale and Post-Traumatic Growth Scale, along with standardized guidance language to prevent bias; The research team conducted weekly case reviews and reinforcement sessions to ensure consistency in intervention delivery. The initial training session is 90 min long, with subsequent weekly sessions lasting 40 min.

#### Sample size calculation

Sample size calculation is based on the results of a randomized controlled trial conducted by Zhang et al.^27^ involving stroke patients. The study findings indicate that stroke patients receiving psychological intervention achieved a SWB score of 9.87 ± 1.95, while the routine care group scored 8.21 ± 2.11^27^. Based on this effect size, with a test power (Power = 1 − β) of 90% and a two-tailed significance level α = 0.05, sample size estimation for an independent samples t-test was performed using G*Power 3.1 software (version 3.1.9.7). Calculations indicated a required sample size of 54 subjects per group. For a total sample size of 108 subjects. Considering potential attrition (estimated at approximately 20%), the final enrollment target was set at 132 patients, who were randomly assigned in a 1:1 ratio to the intervention and control groups, with 66 subjects in each group.

#### Constructing an intervention program

The preliminary draft of the intervention program was formed through the results of the preliminary status survey combined with the literature review and the study of the PERMA model theory. To ensure the scientificity and feasibility of the intervention protocol, seven experts were invited to consult through face-to-face meetings, and the entries and contents of the protocol were improved and modified according to the experts’ suggestions. In order to ensure the practicality and effectiveness of the program, 10 young and middle-aged stroke patients who met the inclusion and exclusion criteria were selected for pretesting, and the problems that occurred in the pretest were analyzed and revised to form the final draft of the PERMA model psychological intervention program.

#### Implementation method

Due to the prolonged functional recovery period following stroke, the average hospitalization duration in the Department of Rehabilitation Medicine is 30 days. Therefore, all interventions in this study were delivered in person during the inpatient period. The control group received standardized routine rehabilitation therapy and nursing care according to the Clinical Practice Guidelines for Stroke Rehabilitation. The intervention group received the PERMA-based intervention program in addition to the control group’s regimen.

##### Control group

The control group received conventional rehabilitation treatment and care, including stroke disease knowledge education, prevention of common post-stroke complications, placement of an anti-spasticity position, rehabilitation exercise, medication instruction, rehabilitation compliance education, dietary instruction, psychological care, and discharge instruction. The total planned contact time over 4 weeks was approximately 3.3 h: 20-min disease knowledge education (once/week, 4 sessions total), 30-min rehabilitation exercise guidance (twice/week, 8 sessions total), integrated guidance on complication prevention, anti-spasticity positioning, medication, diet, and rehabilitation compliance (about 30 min total, embedded in daily care), ad-hoc psychological care (≤ 10 min/session, average 2 sessions), and one-time discharge instruction (10 min).

##### Intervention group

The intervention group received conventional rehabilitation care supplemented by a PERMA-based psychological intervention. The intervention was delivered through eight structured individual sessions (30–45 min each) over a 4-week period, with intervals of 2–4 days between sessions to facilitate patient engagement and reflection. Sessions were conducted one-on-one by two trained graduate students, under the supervision of a research team that included a nationally certified Level-2 psychological counselor and rehabilitation specialists. To accommodate individual patient characteristics and needs, the intervention protocol permitted certain flexible adjustments. These included modifying the sequence of topics or extending session duration when deemed beneficial—for instance, for younger patients (aged 18–44) with higher pre-stroke income levels who might benefit from more in-depth discussion. Additionally, communication strategies were adapted for patients with specific impairments, such as using gestures, writing, or typing for those with dysarthria, and integrating vocalization exercises with positive emotional reinforcement. The specific intervention plan is shown in Table 1.

**Table 1:** Psychological intervention program for young stroke patients in the PERMA model.

Time (Session)	Subject	Intervention content (Fixed Structure: 5-min Icebreaker + Core + Summary + Homework)	Suggested guidance language	Duration (min)	Homework
1st	Understanding stroke	(1) Self-introduction of the subject group; introduction of the department staff and environment (2) Explain the knowledge about stroke and rehabilitation to enhance the patient’s confidence in the rehabilitation treatment of the disease (3) Collect information about the patient, including the patient’s basic information, disease information, psychological status, the degree of harmony with family members, and the degree of support from family members for the patient (4) Establish a trusting relationship with the patient and leave contact information, such as a telephone number and WeChat, with each other. (e.g., patients with language dysfunction can communicate with each other through gestures, writing, or typing)	(1) Do you know about stroke? (2) You can tell me what questions you have about stroke rehabilitation (3) I will be happy to help you if you are confused about the psychological aspects	40	None
2nd	Discovering positive psychological qualities (P)	(1) Use the intake talk technique to understand the psycho-emotional state of the patient (2) Analyze the psychological problems caused by the stroke for the patient and tap into the positive aspects of things (3) Use non-verbal communication such as eye contact and touch to empathize with the patient and give encouragement and emotional support (4) Encourage patients to share positive and happy things about themselves to tap into their positive psychological qualities (5) For patients aged 18–44 years old with a higher monthly income before the disease, increase the communication time or frequency appropriately according to the talks	(1) You can think of me as your friend (a patient of similar age) or your junior (an older patient) and share your happy things with me (2) It is reasonable for you to have such thoughts, and I can understand your feelings (3) I can see that you are in a good state, for example, you just mentioned…, which is very positive	35	Record one positive quality of yourself each day
3rd	Cultivate positive emotions (P)	(1) Start the interview around the concept and role of positive emotion and talk about the benefits of positive emotion and optimism for the disease (2) Cultivate positive thinking and a positive attitude towards stroke events. Recommend watching movies with positive themes, such as “How Steel is Made” and “When Happiness Comes Knocking”, so that patients can cooperate with treatment and rehabilitation with positive emotions and increase the initiative of participation (3) Provide patients with the opportunity to watch the stroke promotional film “Time = Brain” and stroke rehabilitation case videos (4) Give recognition and praise to patients for their positive emotions (5) For patients with dysarthria, practice pronunciation and mobilize positive emotions through vocalization	(1) If you are willing to share, I would love to hear some positive and happy things about yourself (2) Think back to a time when you had a difficult event in your life, and let’s try to analyze it from the positive side (3) According to the stroke propaganda film, people can recover well with active treatment and rehabilitation exercises	30	Watch one positive video (provided by the team) during hospital rest time and share feelings next session
4th	Gratitude exercise (P)	(1) Talk about the positive effects of learning to be grateful for improving emotional states and enhancing the sense of well-being in life (2) Focus on the events in life and during hospitalization for which one needs to be grateful as an entry point, and guide the patient to recall them together (3) Encourage patients to pay attention to the good and happy things in their lives and record three good things that happen every day before going to bed in the form of a diary or a circle of friends, preferably for 21 days, or the patient can dictate them and the family can record them for them	(1) You think of things and people that have touched you in the past or during your hospital stay, and please tell us if it is convenient to share (2) You can write a letter of appreciation, edit a WeChat message, or record a voicemail and send it to the person you appreciate (3) You can keep a diary, send a WeChat friend circle, or any other form of record the happy and beautiful things that happen to you every day	45	Record three grateful things daily for 21 days
5th	Engagement exercise (E)	(1) Explain the definition and positive effects of the “flow” state (2) According to the rehabilitation needs of the patient, the rehabilitation therapist will assess the patient’s condition and then perform rehabilitation activities together under the guidance of the rehabilitation therapist. Let the patient engage in positive emotions and experience a sense of well-being. For example, practice rehabilitation exercises for hand functions such as “picking up beans, screwing nuts, and buttoning” (3) Discuss with patients their hobbies and interests. Combine the patient’s condition and hobbies to develop activities. For example, playing chess, reading books, listening to music, etc. After the patient enters the state of “flow”, the patient’s attention is distracted from the disease and the body and mind are relaxed, which is conducive to positive psychological changes	(1) We can communicate about what hobbies or things you are more interested in (2) How do you feel when we develop rehabilitation activities and do rehabilitation exercises together? You can communicate with me anytime if you have any ideas or suggestions (3) Now imagine that you are at home, sitting in your study reading your favorite book and listening to your favorite song	35	Practice the “flow” activity for 15 min/day during hospital rehabilitation time
6th	Foster positive interpersonal relationships (R)	(1) Talk about positive communication skills and how good communication promotes interpersonal relationships (2) Communicate around interpersonal relationships, give examples of the physical and psychological benefits of good interpersonal relationships, and guide patients to realize the importance of interpersonal relationships (3) Conduct role plays so that patients can appreciate positive communication and experience the joy of interaction (4) Play small games with family members, patients, and friends, such as “finger catching”, if the rehabilitation physician’s assessment of their condition permits (5) Invite stroke patients who have recovered well to communicate with each other	(1) Talk about someone you like or something tough or happy you experienced together (2) I just heard you discussing with a patient lower-limb exercises; it’s good to communicate and learn more (3) XXX is in bed, he is cheerful, optimistic, and engaged in positive rehabilitation exercises; his recovery is good; you can communicate more freely	40	Practice one positive communication skill with family members or medical staff weekly
7th	Perception meaning (M)	(1) Discuss with patients the meaning of life, the meaning of living, the meaning of loved ones, and the meaning of health, and guide them to establish a correct view of meaning. A positive view of meaning will enable patients to develop a sense of mission and direction (2) Discuss the importance of each person’s role in the family and the meaning of that role to loved ones, friends, and society (3) Stimulate the patient’s sense of responsibility to loved ones and society (4) Instruct patients to deal with the ups and downs of life with optimism. As in the case of stroke events, learning to grow in adversity is also the meaning of life (5) Guide patients to self-evaluate and talk about their interpretation of meaning	(1) Talk about something meaningful in your life or work (2) I see that your family members are very patient with you; they need you very much, and you have a very important meaning to them (3) In fact, everyone’s life will encounter some unsatisfactory things, so believe that the rainbow will appear after the storm	35	Write a short essay on “my post-stroke life meaning” (completed during hospitalization)
8th	Experience the achievement (A)	(1) Conduct an interview with the patient about achievement and explain the positive psychological effects of achievement (2) Develop a “rehabilitation prescription” with the patient and, under the guidance of the rehabilitation therapist, help the patient achieve a sense of accomplishment through functional rehabilitation exercises (3) Set achievable goals and encourage the patient to use their strengths under the guidance of the rehabilitation therapist to gain a sense of accomplishment. For example, dressing and undressing by themselves, eating by themselves, going to the bathroom, etc. within the limits of their ability (4) Promptly acknowledge the patient’s achievements and give recognition and praise even for the accomplishment of small goals	(1) I saw you exercising on your own in bed; that’s great! (2) The rehabilitation physician even complimented you on your progress. You are doing great; keep up the good work! (3) Rehabilitation is very crucial in the early stages, and as long as you stick to it every day, a virtuous cycle will be formed (4) What do you have in mind for the future? Now is the time for you to plan	40	None

The intervention was initiated within 3 days of admission, comprising 8 structured face-to-face sessions spaced 2–4 days apart. The mean ± standard deviation session duration was 36.3 ± 4.5 min (range: 30–45 min), with a mean ± standard deviation total contact time of 305.6 ± 36.0 min (range: 280–340 min, approximately 5.1 h). All 66 patients completed all 8 sessions, achieving a 100% completion rate. Total contact time showed minimal variation (≤ 5% deviation from the planned 290 min).

To ensure intervention integrity and individualization, fixed components included session sequence, PERMA themes, 30–45-min duration, and structure; tailoring was based on age/income, language function, and rehabilitation progress (detailed in Table 1). Fidelity was maintained via a standardized manual, checklist-based assessment, pre-intervention training, weekly supervision, and on-site monitoring (deviation rate ≤ 5%).

The additional contact time in the intervention group was strictly allocated to structured content on PERMA themes (e.g., positive emotion cultivation, flow experiences, relationship enhancement, life meaning exploration, and achievement experiences), rather than additional casual attention or repetitive routine care. This ensured that the intervention’s specific effects could be distinguished from non-specific biases.

#### Randomization, allocation concealment, and blinding

This study employed a single-blind, 1:1 simple randomization parallel-group design, implementing blinding for participants, outcome assessors, and data analysts. Specific implementation details are as follows:

Random sequence generation: A statistical expert (not part of the research team) generated a simple randomization sequence using SPSS 25.0 software, producing 132 random numbers (corresponding to the planned enrollment of 132 patients). Each random number was assigned to either the “Intervention Group” or “Control Group” in a 1:1 allocation ratio, ensuring balanced sample sizes between groups. The generated random sequence was printed, archived, and sealed in an opaque envelope.

Allocation concealment: A departmental research assistant (not part of the research team) wrote the group assignment information corresponding to each random number into opaque, sealed envelopes labeled with unique identification codes. These envelopes were stored in numerical order. After a patient met the inclusion criteria and signed the informed consent form, the research assistant sequentially opened the envelope corresponding to that patient.

Participants: All patients were informed that “this study evaluates the effectiveness of rehabilitation support, and both groups will receive personalized rehabilitation guidance”. The terms “intervention group” and “control group” were not disclosed, and the duration of each single intervention session (30–45 min) was standardized for both groups.

Scale Assessors: Intermediate-level rehabilitation nurses assessed subjective well-being (SWB) and posttraumatic growth (PTG) using standardized protocols, avoiding discussions about intervention content or group assignments with patients.

Data Analysts: Data entry used coded designations “Group A” and “Group B,” with dual entry and cross-verification. The coding-group mapping relationship was revealed after statistical analysis completion.

Minimizing Performance and Detection Bias: Roles were separated: intervention implementation, outcome assessment, and data analysis were performed by distinct personnel with no overlapping responsibilities.

Non-Blinded Roles and Bias Control: The intervention group received PERMA-based psychological intervention, while the control group received routine rehabilitation therapy. Therefore, intervention providers could not be blinded.

#### Adverse event monitoring

In accordance with CONSORT Item 19, adverse events (AEs) were systematically monitored throughout the study. Adverse events were defined as any negative physiological response (e.g., increased physical pain, worsening neurological function) or psychological response (e.g., heightened anxiety, emotional distress) potentially related to the intervention or routine rehabilitation. Monitoring methods include: Rehabilitation physicians assessing patients’ physical and psychological suitability for intervention prior to treatment; Intervention providers inquiring about immediate discomfort post-treatment; Systematic inquiry into delayed reactions during 1-month and 3-month follow-up periods.

### Data collection

Assessments were made by scale before the intervention, immediately after the intervention, 1 month after the intervention, and 3 months after the intervention. Inpatients were assessed face-to-face in quiet, private spaces (rehabilitation ward/health education room) with minimal distractions; discharged patients underwent face-to-face assessments at the hospital’s follow-up clinic (consistent with inpatient settings) or telephone/video (WeChat) assessments in noise-free environments. To minimize inter-rater variability, all patients were assessed at all time points (baseline, post-intervention, 1-month, and 3-month follow-up) by the same two rehabilitation nurses. The assessors maintained consistent assessment procedures and standardized language during both in-person and telephone evaluations.

Assessment and follow-up timepoints (baseline/immediately post-intervention/1 month/3 months), including permitted access windows, employ standardized visit windows to minimize longitudinal comparison bias:—Baseline (T0): Within 72 h (± 1 day) of patient admission, prior to PERMA intervention, collect pre-intervention SWB, PTG, and baseline characteristic data; Immediately post-intervention (T1): 4 weeks (8 sessions) (± 1 day); 1-month follow-up (T2): 30 days (± 7 days) after T1, assessed via in-person hospital follow-up clinic visit or telephone/WeChat video call.3-month follow-up (T3): 90 days (± 7 days) after T1, assessed via in-person hospital follow-up clinic visit or telephone/WeChat video call.

Both SWB and PTG are self-report instruments, with all items expressed verbally (excluding visual stimuli or items dependent on written content). This characteristic makes them suitable for telephone/video assessment. Existing research has confirmed the equivalence of telephone administration^28,29^, demonstrating no systematic bias in scoring collection across different assessment modalities. To ensure equivalence with in-person assessments, this study employed trained rehabilitation nurses who uniformly guided responses and recorded patient answers in real time.

### Evaluation tools

1. General information questionnaire

The investigator’s own design included two sections: demographic and sociological information (gender, age, marital status, education, occupation, place of residence, monthly income, type of health insurance, smoking, alcohol consumption, primary caregiver) and disease-related information (comorbid chronic diseases, number of strokes, family genetic history [defined as stroke history in first-degree relatives: parents, siblings]).

(2) Subjective well-being (SWB)

The subjective well-being (SWB) Scale (Index of Well-Being) was originally developed by Campbell et al.^30^ and translated into Chinese by Fan Xiaodong^31^. It consists of two parts: the Overall Affect Index Scale (8 items) and the Overall Life Satisfaction Questionnaire (1 item), both rated on a 7-point Likert scale. The total score is calculated by summing the mean score of the Overall Affect Index Scale and the score of the Life Satisfaction Questionnaire (weighted by 1.1), ranging from 2.1 to 14.7, with 2.1–6 indicating low SWB, 6.1–10 moderate SWB, and 10.1–14.7 high SWB. This scale has demonstrated good psychometric properties in prior Chinese populations (Cronbach’s α = 0.85–0.91)^31^.

(3) Post-traumatic growth (PTG)

The post-traumatic growth inventory (PTGI) was developed by Tedeschi and Calhoun^11^ and adapted into Chinese by Wang Ji et al.^32^. It comprises 5 dimensions and 20 items: relationships with others (7 items), new possibilities (5 items), personal strength (4 items), spiritual change (1 item), and appreciation of life (3 items). Each item is rated from 0 to 5, with total scores ranging from 0 to 100; higher scores indicate higher levels of post-traumatic growth. The psychometric properties of the Chinese version have been well documented, with a total scale Cronbach’s α of 0.87 and subscale α coefficients ranging from 0.61 to 0.87^32^.

### Statistical methods

All data were statistically analyzed using SPSS 25.0 software, with a two-tailed *P* 0.05 considered statistically significant.

1. Categorical variables (nominal level, e.g., gender, marital status, smoking status, comorbid chronic diseases) were described as frequencies and percentages (n, %), and intergroup comparisons were performed using the chi-square test.
2. Ordinal variables (ordinal level, e.g., educational background, monthly income grade, number of strokes) were presented as frequencies and percentages (n, %), and intergroup comparisons were conducted using the rank-sum test (Mann–Whitney U test for two independent groups).
3. Continuous variables (continuous level) were first tested for normality:
  Normally distributed variables (e.g., age, subjective well-being score, post-traumatic growth score) were expressed as mean ± standard deviation ($\overline{x}$ ± s), and intergroup comparisons at the same time point were performed using the independent samples t-test; Non-normally distributed continuous variables (if any) were presented as median (interquartile range) [M (IQR)], with intergroup comparisons using the Mann–Whitney U test.
4. Repeated measures analysis of variance (ANOVA) was used to compare changes in continuous outcome variables (SWB and PTG scores) across different time points (pre-intervention, post-intervention, 1-month follow-up, 3-month follow-up) and to analyze time effects, between-group effects, and interaction effects. Normality was confirmed via Shapiro–Wilk tests (all *P* values > 0.05), while homogeneity of variance–covariance matrices was verified using Box’s M test (all *P* values > 0.05).

## Results

### Study population

**Fig. 1:**
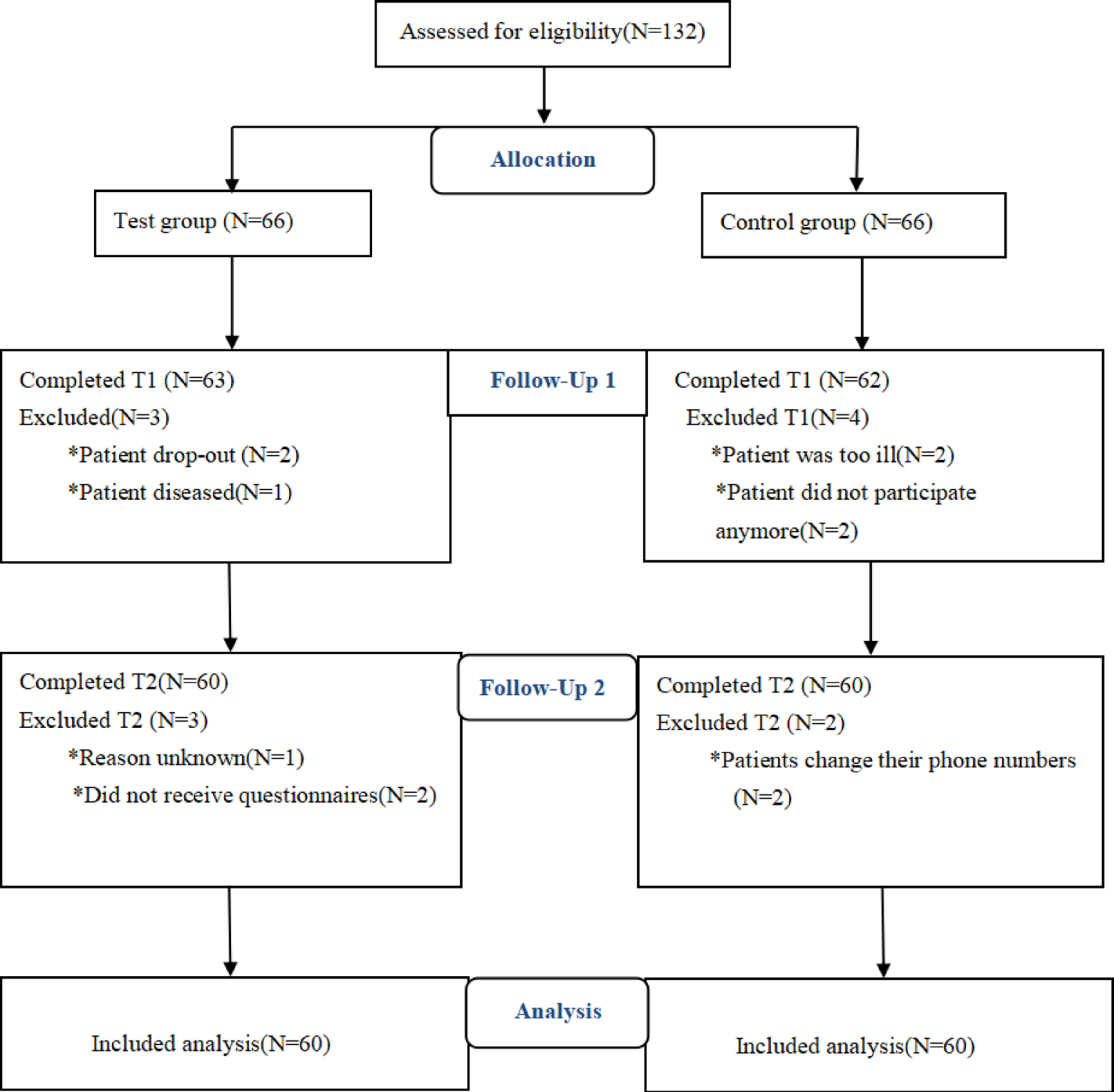
Flowchart of sample selection.

A total of 132 middle-aged and young stroke patients meeting inclusion and exclusion criteria were randomly assigned to each group (n = 66). During the 4-week intervention period, all patients completed all 8 treatment sessions. No adverse events were reported or observed in either the control group (receiving conventional rehabilitation therapy) or the intervention group (receiving conventional rehabilitation therapy + PERMA intervention). Dropouts occurred during the post-intervention follow-up: 12 patients were lost to follow-up at the 1-month and 3-month visits (dropout rate: 9.09%). Ultimately, 60 complete cases per group were included in the analysis (see Fig. 1).

### Comparison of general information between the two groups of patients

The differences between the general data of the two groups of patients were not statistically significant (*P* > 0.05) and were comparable, as shown in Table 2.

**Table 2:** Comparison of general information between the two groups of patients (N = 120).

Projects	Classification	Control group (60)	Intervention group (60)	Test statistic (χ^2^/z value)	*P* value
Gender	Male	42	46	0.682^a^	0.409
	Female	18	14		
Age (years)	18–44	9	10	0.063^a^	0.803
	45–64	51	50		
Marital status	Married	58	59	0.342^a^	0.599
	Unmarried	2	1		
Education	Primary	7	6	− 0.372^b^	0.710
	Middle school	15	18		
	High school	23	16		
	University	15	20		
Occupation	Employee	38	42	4.969^a^	0.174
	Businessmen	7	11		
	Farmer	10	3		
	Unemployed	5	4		
Place of residence	Shenzhen	47	43	0.711^a^	0.399
	Non-Shenzhen	13	17		
Monthly income (¥)	< 1000	11	5	− 1.278^b^	0.201
	1000–2999	3	2		
	3000–4999	14	16		
	5000–10,000	20	22		
	> 10,000	12	15		
Insurance type	Urban insurance	45	52	5.768^a^	0.056
	Rural insurance	14	5		
	Self-funded	1	3		
Smoking	No smoking	32	27	0.834^a^	0.361
	Smoking	28	33		
Drinking	No drinking	37	30	1.656^a^	0.198
	Drinking	23	30		
Comorbid chronic diseases	No	15	16	0.043^a^	0.835
	Yes	45	44		
Number of strokes	Primary	42	50	− 1.655^b^	0.098
	Secondary	16	8		
	Tertiary	2	2		
Primary caregiver	Spouse	47	45	0.710^a^	0.701
	Relatives	2	4		
	Caregiver	11	11		
Genetic history	No	28	23	0.853^a^	0.356
	Yes	32	37		

Note: ^a^χ^2^ value corresponds to the chi-square test (for categorical variables).

^b^z value corresponds to the Mann–Whitney U rank-sum test (for ordinal variables).

### Comparison of baseline data scores between the two groups of patients before the intervention

The baseline data scores were not significantly different between the two groups before the intervention (*P* > 0.05) and were comparable, as detailed in Table 3.

**Table 3:** Comparison of baseline data scores between the two groups of patients before the intervention (points, $\overline{\mathrm{x}}$ ± s).

Projects	Group	Average value	*t* value	*P* value
Subjective well-being (SWB)	Control group (60)	7.53 ± 2.885	− 0.459	0.647
	Intervention group (60)	7.78 ± 2.978		
Post-traumatic growth inventory (PTGI)	Control group (60)	43.81 ± 15.977	− 0.419	0.676
	Intervention group (60)	45.34 ± 22.944		

### Comparison of SWB and PTG scores between the two groups

**Fig. 2:**
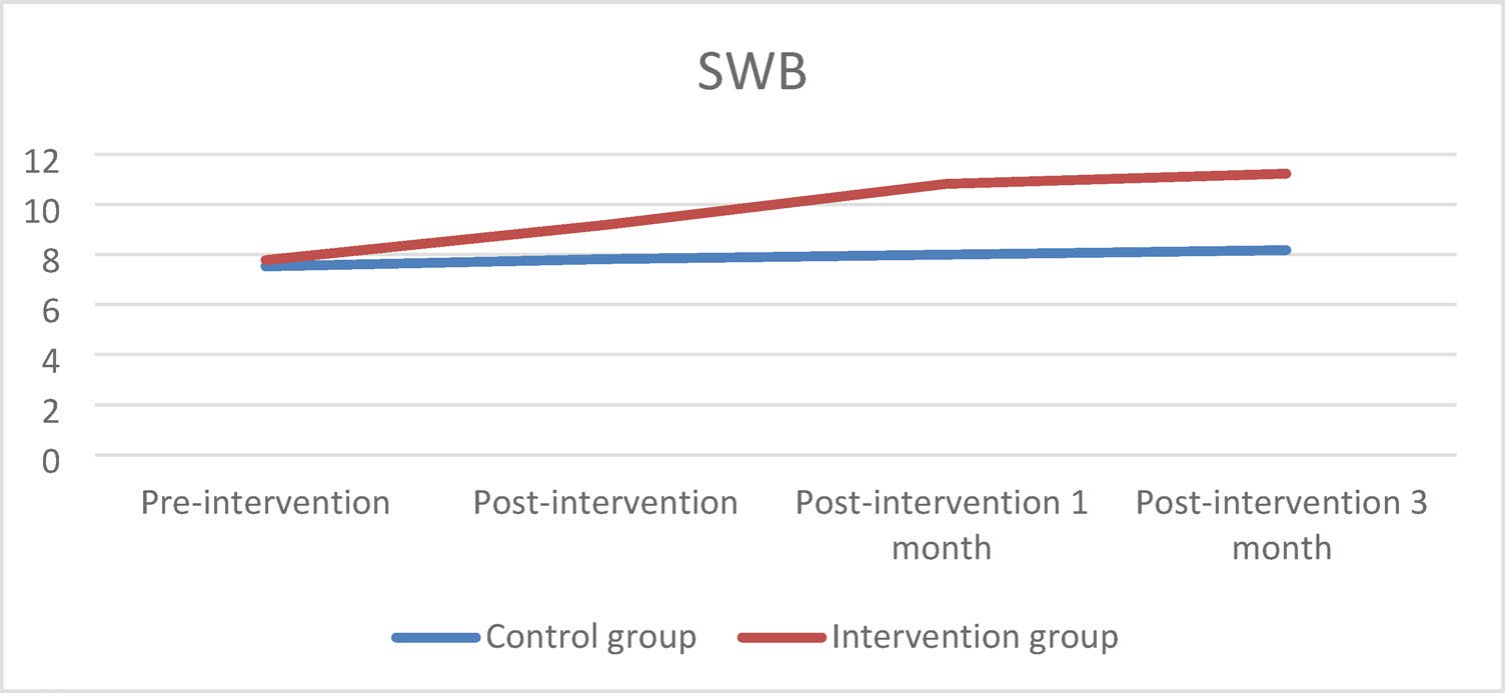
Graph of SWB scores over time. *Note*: Data are presented as mean ± standard deviation (SD). SWB scale score range: 2.1–14.7 (2.1–6 = low SWB, 6.1–10 = moderate SWB, 10.1–14.7 = high SWB). Control group (n = 60); Intervention group (n = 60).

**Fig. 3:**
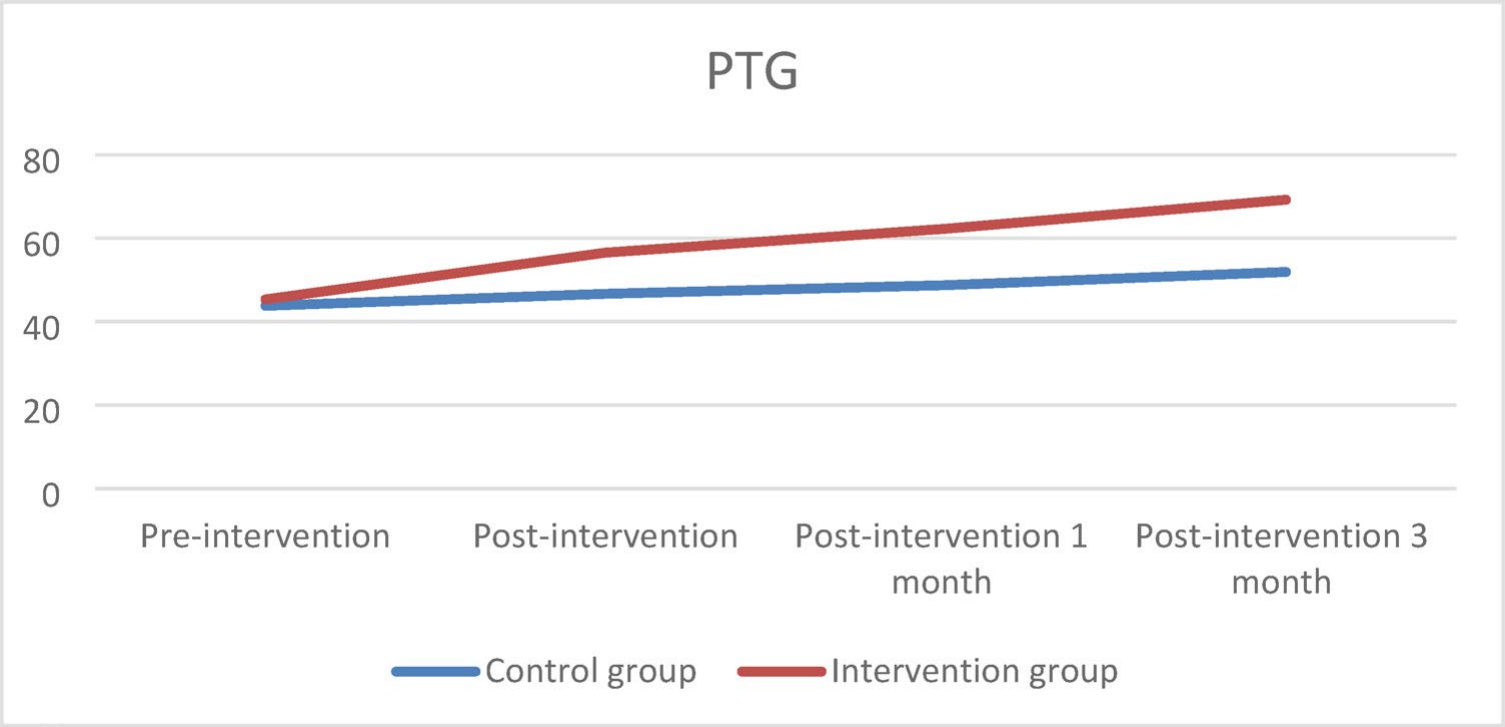
Graph of PTG scores over time. *Note*: Data are presented as mean ± standard deviation (SD). PTG scale score range: 0–100 (higher scores indicate higher levels of post-traumatic growth). Control group (n = 60); Intervention group (n = 60).

The comparison of SWB and PTG scores at each time point after the intervention was statistically higher in the intervention group than in the control group (*P* < 0.05), as shown in Table 4. The subjective well-being scores of the patients in both groups changed over time and showed an increasing trend, but the increase in the intervention group was more obvious than that in the control group, as shown in Fig. 2. The post-traumatic growth levels of patients in both groups showed an increasing trend, but the increase was more pronounced in the intervention group than in the control group, as shown in Fig. 3.

**Table 4:** Between-group comparisons of SWB and PTG scores (independent samples t-test).

Outcome measure	Group	Baseline ($\overline{\mathrm{x}}$ ± s)	Post-intervention ($\overline{\mathrm{x}}$ ± s)	1-month follow-up ($\overline{\mathrm{x}}$ ± s)	3-month follow-up ($\overline{\mathrm{x}}$ ± s)
SWB	Control (n = 60)	7.53 ± 2.885	7.82 ± 2.864	8.00 ± 2.838	8.18 ± 2.876
	Intervention (n = 60)	7.78 ± 2.978	9.19 ± 2.952	10.83 ± 2.720	11.24 ± 2.603
t-value		0.459	2.584	5.572	6.099
*P* value		> 0.05	< 0.01	< 0.001	< 0.001
95% CI		− 0.27, 0.43	0.12, 0.82	0.64, 1.39	0.74, 1.49
PTG	Control (n = 60)	43.81 ± 15.977	46.68 ± 15.873	48.75 ± 15.274	51.92 ± 15.397
	Intervention (n = 60)	45.34 ± 22.944	56.55 ± 22.808	62.28 ± 21.719	69.23 ± 20.086
t-value		0.419	2.750	3.948	5.300
*P* value		> 0.05	< 0.01	< 0.001	< 0.001
95% CI		− 0.28, 0.44	0.15, 0.85	0.36, 1.07	0.59, 1.34

### Comparison of repeated measures ANOVA for SWB and PTG

A repeated measures ANOVA was performed on the subjective well-being scores of the two groups at different times, and the time effect (*F* = 616.854, *P* < 0.05), the between-group effect (*F* = 12.452, *P* < 0.05) and the interaction effect between the two groups (*F* = 47.246, *P* < 0.05) were all statistically significant, as shown in Table 4. The post-traumatic growth scores of the two groups at different times were analyzed by repeated measures ANOVA, and the time effect (F = 885.384, *P* < 0.05), between-group effect (F = 10.337, *P* < 0.05) and interaction effect (F = 221.684, *P* < 0.05) were all statistically different, as shown in Table 5.

**Table 5:** Repeated-measures ANOVA results for SWB and PTG scores.

Outcome measure	Effect type	F-value	*P* value
SWB	Time effect	1309.157	< 0.001
	Between-group effect	13.354	< 0.001
	Time by group interaction effect	635.436	< 0.001
PTG	Time effect	885.384	< 0.001
	Between-group effect	10.337	< 0.01
	Time by group interaction effect	221.684	< 0.001

Repeated-measures ANOVA analyzes score changes across 4 time points (baseline, post-intervention, 1-month follow-up, 3-month follow-up). Time Effect = within-group differences across time; Between-Group Effect = overall differences between groups; Interaction Effect = differential change trends between groups.

### Effect sizes

For SWB, Cohen’s d (intervention vs. control) was 0.47 (95% CI 0.11, 0.82) at post-intervention, increasing to 1.11 (95% CI 0.74, 1.49) at 3-month follow-up. For PTG, effect sizes were 0.50 (95% CI 0.15, 0.85) at post-intervention and 1.02 (95% CI 0.59, 1.34) at 3 months. According to conventional benchmarks^33^, these represent medium effects at post-intervention and large effects at follow-up.

## Discussion

This randomized controlled trial demonstrated that a rehabilitation-aligned PERMA-based psychological intervention, delivered over 4 weeks, resulted in significantly greater improvements in subjective well-being (SWB) and post-traumatic growth (PTG) among young and middle-aged stroke patients, compared to conventional rehabilitation care alone. The benefits were observed immediately after the intervention and were sustained at the 1-month and 3-month follow-up. The findings of this study are consistent with the positive outcomes reported in other studies examining the psychological adjustment of stroke survivors^34^; timely psychological intervention during the rehabilitation period can enhance the effectiveness of rehabilitation.

### Implications of the findings of this study for clinical practice

The findings carry several practical implications for rehabilitation teams working with young and middle-aged stroke survivors.

First, intervention is feasible within inpatient rehabilitation settings. With eight 30–45-min sessions delivered over 4 weeks (total ~ 5.1 h), the PERMA protocol fits within typical rehabilitation hospitalization periods (average 30 days in this setting) without requiring additional staff. Implementation required only two trained graduate students supervised by a psychological counselor—a model that could be adapted using rehabilitation nurses or occupational therapists after brief training (initial 90-min session plus weekly 40-min reinforcement).

Second, the structured yet flexible format (Table 1) allows tailoring to individual patient characteristics. Specific adaptations—such as adjusting session duration for younger patients (18–44 years) with higher pre-stroke income, using non-verbal communication for patients with dysarthria, or modifying rehabilitation activities based on functional level—demonstrate that the PERMA model can be integrated with ongoing physical therapy rather than delivered as a standalone psychological intervention. This integration is particularly valuable because it normalizes psychological support as part of rehabilitation rather than as a separate “treatment”.

Third, the sustained effects at 3-month follow-up suggest that the skills learned (e.g., gratitude recording, flow engagement, meaning reframing) generalized beyond the inpatient period. Clinically, this implies that brief inpatient interventions can produce durable changes in well-being, potentially reducing post-discharge depression risk and improving long-term rehabilitation adherence. The 21-day gratitude exercise (Session 4) aligns with habit-formation literature, which may explain the persistence of benefits.

Fourth, for rehabilitation teams, the PERMA framework offers a shared vocabulary for addressing psychological needs that is non-pathologizing and strengths based. Rather than focusing on deficits (e.g., “depression screening”), the intervention reframes patient struggles as opportunities for growth. This may reduce stigma and increase patient engagement with psychological support.

Finally, resource considerations: The intervention required approximately 1.7 additional contact hours compared to the control group (5.1 vs. 3.3 h). While this extra time was associated with superior outcomes, future implementation should test whether similar effects can be achieved with a dosed-down version (e.g., 6 sessions) or delivered by rehabilitation nurses rather than dedicated interventionists to improve scalability.

### Interpretation of findings and hypothesized mechanisms

The positive outcomes align with previous research applying positive psychology frameworks in medical populations^7,19,27^. However, it is important to note that this study did not include process measures (e.g., changes in positive affect, perceived support) or mediator analyses. Therefore, the following explanations for why the intervention might have worked remain speculative and are based on the intervention’s design and theoretical background.

We hypothesize that the integration of PERMA elements with concrete rehabilitation activities could have been a contributing factor. For instance, by linking discussions of “Achievement” (A) to specific, incremental functional goals (e.g., self-dressing), the intervention may have helped patients reframe rehabilitation progress as a source of accomplishment rather than just a medical necessity. Similarly, focusing “Meaning” (M) discussions on patients’ ongoing roles within their families might have addressed identity concerns common after stroke. The structured yet flexible format (Table 1) may have facilitated a supportive therapeutic relationship, which is a common factor associated with positive outcomes in psychosocial interventions.

It is also plausible that the observed benefits stem from a combined effect of addressing multiple well-being domains (positive emotion, engagement, relationships, meaning, achievement) simultaneously, as suggested by the PERMA theory^17^. Future research should directly measure these proposed mechanisms to test these hypotheses.

### Consideration of alternative explanations and study limitations

Outcome Reporting and Selective Analysis: This study focused on the primary psychological outcomes (SWB, PTG) as pre-specified. However, a comprehensive comparison with the initial trial registry is warranted in future full reports to ensure complete transparency. Furthermore, our analysis was conducted on a complete-case basis, excluding the 12 participants (9.09%) lost to follow-up. While this dropout rate was relatively low and balanced between groups, the use of complete-case analysis assumes data are missing completely at random, which may not hold true. This approach could introduce bias and typically overestimates treatment effects compared to intention-to-treat analyses with appropriate imputation methods^35^.

Blinding and measurement bias: As a psychological intervention trial, full blinding of participants and intervention providers was not feasible. Although outcome assessors were blinded, the primary outcomes were self-reported questionnaires (SWB and PTG). The knowledge of receiving a novel, positive psychology intervention may have influenced participants’ responses due to social desirability or expectation effects^36^. Similarly, intervention providers’ enthusiasm could have unintentionally biased their delivery. While we trained assessors to use standardized language, the risk of detection and performance bias for subjective endpoints remains a notable limitation.

Intervention fidelity and potential confounders: We implemented several strategies to ensure intervention fidelity, including a standardized manual, staff training, and supervision. However, we did not employ quantitative fidelity measures (e.g., session audio recording and independent rating) or formally assess therapeutic alliance, which are recommended to verify that the intervention was delivered as intended and to distinguish specific from non-specific therapeutic factors^37^. Most significantly, the intervention group received substantially more total contact time (~ 5.1 h) than the control group (~ 3.3 h). Therefore, the observed benefits cannot be disentangled from the potential effects of additional attention and supportive interaction, a key non-specific factor common in psychotherapy research.

Generalizability: Our sample was restricted to young and middle-aged inpatients with moderate-to-severe ischemic stroke (mRS ≥ 3) in a single rehabilitation center. The results may not extend to older patients, those with mild stroke, hemorrhagic stroke, or individuals in community settings, who may have different psychological profiles and recovery trajectories.

Mechanistic Uncertainty: The study was not designed to test the active mechanisms of the PERMA model. We did not include process measures (e.g., changes in positive affect, resilience, or specific PERMA domain scores) to mediate the observed outcomes. Thus, our discussion of mechanisms remains hypothetical and requires empirical validation in future research with a theory-driven, mediation analysis design.

## Conclusions

Notwithstanding these limitations, this randomized controlled trial provides preliminary, encouraging evidence that a structured, PERMA-based positive psychology intervention, integrated into standard inpatient stroke rehabilitation, is associated with significant and sustained improvements in subjective well-being and post-traumatic growth among young and middle-aged survivors.

Given the constraints related to blinding, self-reported outcomes, and the imbalance in contact time, the results should be interpreted as supporting the potential efficacy and feasibility of the overall intervention package rather than definitively establishing the specific superiority of the PERMA components alone. The positive effect sizes and meaningful score changes justify further investigation.

Future research should prioritize: (1) employing active control conditions (e.g., equal-attention supportive counseling) to isolate the specific effects of PERMA; (2) adhering to intention-to-treat principles with robust methods for handling missing data; (3) incorporating objective or blinded outcome measures where possible and quantitative fidelity assessments; (4) conducting mediation analyses to elucidate causal pathways; and (5) testing the intervention in more diverse stroke populations and healthcare settings to evaluate its broader applicability and effectiveness.

## Data Availability

The datasets used and/or analysed during the current study available from the corresponding author on reasonable request.
